# ﻿Multigene phylogeny, taxonomy, and potential biological properties of *Pseudoroussoella* and *Neoroussoella* species (Roussoellaceae, Dothideomycetes) from Asteraceae weeds in northern Thailand

**DOI:** 10.3897/mycokeys.111.136922

**Published:** 2024-12-17

**Authors:** Zin Hnin Htet, Kevin D. Hyde, Fatimah O. Alotibi, Thilini K. W. Chethana, Ausana Mapook

**Affiliations:** 1 School of Science, Mae Fah Luang University, Chiang Rai 57100, Thailand; 2 Center of Excellence in Fungal Research, Mae Fah Luang University, Chiang Rai 57100, Thailand; 3 Innovative Institute for Plant Health, Zhongkai University of Agriculture and Engineering, Haizhu District, Guangzhou 510225, China; 4 Botany and Microbiology Department, Faculty of Science, King Saud University, Riyadh, 11451, Saudi Arabia

**Keywords:** 2 new species, antibacterial properties, Ascomycota, *Bidenspilosa*, *Chromolaenaodorata*, new host record

## Abstract

In our study, dead stems of two Asteraceae species (weeds) were collected from northern Thailand. Both morphology and multigene phylogeny were used to determine the identity of the taxa. Maximum likelihood and Bayesian inference analyses of combined LSU, SSU, ITS, *tef1-α* and *rpb2* data revealed two new species *Pseudoroussoellabidenticola*, and *Neoroussoellachromolaenae* with one new host record of *N.entadae*. Preliminary investigation into antibacterial properties revealed that our three isolates inhibited the growth of *Bacillussubtilis*, *Escherichiacoli*, and *Staphylococcusaureus*. Additionally, we present updated phylogenetic trees for Roussoellaceae, accompanied by detailed descriptions and illustrations of the three identified species.

## ﻿Introduction

Asteraceae species exhibit a widespread distribution from polar to tropical regions (Xu et al. 2017). Many of these species hold economic significance, while others are categorized as weeds (Jansen and Palmer 1987; Katinas et al. 2007; Karlsson et al. 2008). In Thailand, numerous invasive weeds have an extensive spread, with *Bidenspilosa* and *Chromolaenaodorata* being prevalent at roadsides, disturbed areas, and agricultural lands (Zungsontiporn et al. 2020). Mapook et al. (2020) studied the fungal diversity in *Chromolaenaodorata* and provided a global checklist of fungi associated with this plant. Moreover, the information of fungi associated with *Bidenspilosa* was provided in previous studies (Abdou et al. 2010; Guatimosim et al. 2015; Zhang et al. 2018; Li et al. 2020; Htet et al. 2024). However, more knowledge is still needed about the diversity of fungi in Asteraceae plants to understand the fungi associated with this plant family. Moreover, the diversity of fungi in these two invasive weeds in Thailand is being further explored.

Roussoellaceae was introduced by Liu et al. (2014) based on morphology and LSU, ITS, *tef1-α* and *rpb2* sequence data. Members of Roussoellaceae can be found as saprobes and human pathogens (Ahmed et al. 2014; Liu et al. 2014; Mapook et al. 2020; Hyde et al. 2023; Wu et al. 2023). Currently, there are 12 genera in Roussoellaceae, viz., *Appendispora*, *Cytoplea*, *Elongatopedicellata*, *Immorrhia*, *Neoroussoella*, *Pararoussoella*, *Pseudoneoconiothyrium*, *Pseudoroussoella*, *Roussoella*, *Roussoellopsis*, *Setoarthopyrenia*, and *Xenoroussoella* (Wijayawardene et al. 2022; Index Fungorum www.indexfungorum.org).

*Neoroussoella* was introduced by Liu et al. (2014) to accommodate a saprobic roussoella-like taxon with the type species *N.bambusae*. The sexual morphology of *Neoroussoella* is defined by immersed ascostromata beneath a clypeus or epidermis, appearing as black, dome-shaped, or flattened ovoid structures on the host surface. The asci are bitunicate and cylindrical, while the ascospores are brown or yellowish-brown, ellipsoidal to fusiform, and 2-celled, surrounded by a mucilaginous sheath (Liu et al. 2014). The asexual morphology of *Neoroussoella* is characterized by superficial or immersed pycnidia with annellidic, ampulliform, cylindrical conidiogenous cells, producing hyaline, pale brown, oblong to ellipsoidal conidia, each with two guttules (Liu et al. 2014; Jayasiri et al. 2019). Currently, there are 15 epithets listed in the Index Fungorum (www.indexfungorum.org), viz., *Neoroussoellaalishanensis*, *N.bambusae*, *N.clematidis*, *N.chiangmaiensis*, *N.entadae*, *N.fulvicomae*, *N.heveae*, *N.lenispora*, *N.leucaenae*, *N.lignicola*, *N.magnoliae*, *N.peltophora*, *N.sedimenticola*, *N.solani*, and *N.thailandica*. Recent studies into the genus have been conducted by De Silva et al. (2022) and Hyde et al. (2023).

*Pseudoroussoella* was introduced by Mapook et al. (2020) based on morphology and LSU, SSU, ITS, *tef1-α* and *rpb2* sequence data. The sexual morph of *Pseudoroussoella* species is characterized by globose to subglobose, dark brown to black ascomata with an ostiole, comprised of *textura epidermoidea* to *textura angularis* or *textura intricata* cells, with septate, trabeculate pseudoparaphyses, 8-spored, bitunicate, fissitunicate, cylindrical to clavate asci with a pedicel, and uniseriate, hyaline to pale brown, oval to ellipsoid, 1-septate ascospores bearing a gelatinous sheath (Mapook et al. 2020). Asexual morphs of *Pseudoroussoella* species are distinguished by solitary, superficial, uni-loculate, globose to obpyriform, pycnidial conidiomata with an ostiole, comprised of *textura angularis* cells, annellidic, ampulliform to oblong, hyaline and unbranched conidiogenous cells and pale brown to reddish brown, aseptate conidia with guttules (Mapook et al. 2020). Currently, two species are listed in the Index Fungorum (www.indexfungorum.org).

Some genera from Roussoellaceae, like *Roussoella* and *Neoroussoella*, are recognized for their bioactive secondary metabolites (Takekawa et al. 2013; Phukhamsakda et al. 2018; Chen et al. 2021; Zhong et al. 2021; Sommart et al. 2022). Moreover, the prescreening for antibacterial activity conducted by Mapook et al. (2020) revealed that *Pseudoroussoellaelaeicola* (MFLUCC 17-1483) inhibits the growth of *E.coli*, resulting in a 10 mm inhibition zone. These findings showed that the species of Roussoellaceae are potential organisms for the production of bioactive secondary metabolites.

In this study, we introduce one new species of *Pseudoroussoella* on *Bidenspilosa* (Asteraceae), and one new species with a new host record of *Neoroussoella* on *Chromolaenaodorata* (Asteraceae). We also provide an updated phylogenetic tree for Roussoellaceae, based on a combined dataset of LSU, SSU, ITS, *tef1-α* and *rpb2* sequence data. Further, we explore the potential antibacterial activity of our three isolates and discuss their implications for future discoveries of bioactive compounds.

## ﻿Materials and methods

### ﻿Sample collection, morphological study and isolation

Dead stems from the Asteraceae plants, *Bidenspilosa* and *Chromolaenaodorata*, were collected from northern Thailand. All specimens were brought to the lab in plastic bags labelled with the collection information. Single spore isolation was performed on malt extract agar (MEA) and incubated at 27 °C for 24 hours (Senanayake et al. 2020). The spores were germinated within 24 h using a Motic SMZ 168 Series microscope (Motic Asia, Hong Kong). Germinated spores were transferred to fresh MEA plates. All the detailed morphological characteristics were observed using a Nikon ECLIPSE 80i compound microscope (Nikon, Japan) fitted to a Canon 550D digital camera (Canon, Japan). Tarosoft Image Framework (v 0.9.7) was used to measure photomicrograph structures. Adobe Photoshop CS6 Extended (v 10.0.) was used to edit and prepare photo plates (Adobe system, USA). Forty-day-old cultures were used for molecular studies. Specimens were deposited at the Mae Fah Luang University Herbarium (Herb. MFLU) while living cultures were maintained at Mae Fah Luang University Culture Collection (MFLUCC). Faces of fungi (FoF) and Index Fungorum (IF) numbers were obtained as instructed by Jayasiri et al. (2015) and Index Fungorum (www.indexfungorum.org). Moreover, the species descriptions were submitted to the GMS Microfungi database (Chaiwan et al. 2021).

### ﻿DNA extraction, PCR amplification and sequencing

Fifty-day-old fungal mycelium was scraped off and placed into a 1.5 ml micro-centrifuge tube using a sterile lancet. Genomic DNA extraction was done using the E.Z.N.A.® Tissue DNA Kit (Omega Biotek Inc., Georgia). Polymerase chain reaction (PCR) was used for DNA amplifications, following the protocols of Mapook et al. (2016). The details of PCR primers and protocols are shown in Table 1. The quality of PCR products was confirmed on 1% agarose gels. Purification and sequencing of PCR fragments with the primers mentioned above were carried out at a commercial sequencing provider (Solgent Co., Ltd, Thailand). The newly generated nucleotide sequences were deposited in the GenBank, and accession numbers were obtained (Table 2).

**Table 1.** PCR conditions used in this study.

Gene	Primers		PCR Conditions
	Forward	Reverse	
Large subunit (LSU)	LR0R	LR5	95 °C: 3 min, (94 °C: 30 s, 56 °C: 50 s, 72 °C: 1 min) × 40 cycles 72 °C: 7 min.
Small subunit (SSU)	NS1	NS4	95 °C: 3 min, (94 °C: 30 s, 55 °C: 50 s, 72 °C :1 min) × 40 cycles 72 °C: 7 min.
Internal transcribed spacer (ITS)	ITS5	ITS4	95 °C: 3 min, (94 °C: 30 s, 55 °C: 50 s, 72 °C :1 min) × 40 cycles 72 °C: 7 min.
Elongation factor-1 alpha (*tef1- α*)	EF1-983F	EF1-2218R	95 °C: 3 min, (94 °C: 30 s, 55 °C: 50 s, 72 °C: 1 min) × 40 cycles 72 °C: 7 min.
RNA polymerase II subunit (*rpb2)*	fRPB2-5F	fRPB2-7cR	95 °C: 5 min, (95 °C : 1 min, 52 °C: 2 min, 72 °C: 90 s) × 40 cycles 72 °C: 10 min

**Table 2.** List of taxa, specimens and sequences used in phylogenetic analyses.

Species	Strain numbers	GenBank accession numbers				
		ITS	LSU	SSU	*tef1- α*	*rpb2*
*Neoroussoellaalishanense*	FU31016	MK503816	MK503822	MK503828	–	MN037756
*N.alishanense*	FU31018	MK503818	MK503824	MK503830	MK336182	MN037757
*N.bambusae*	MFLUCC 11-0124 T	KJ474827	KJ474839	–	KJ474848	KJ474856
***N.chromolaenae* sp. nov.**	**MFLUCC 24-0274**	**PQ226190**	**PQ226193**	**PQ226196**	**PQ240621**	**PQ240623**
*N.clematidis*	MFLUCC 17-2061	MT310632	MT214587	MT226700	MT394645	MT394701
*N.entadae*	MFLUCC 18-0243	MK347786	MK348004	MK347893	MK360065	MK434866
***N.entadae***	**MFLUCC 24-0275**	**PQ226191**	**PQ226194**	**PQ226197**	–	**PQ240624**
*N.fulvicomae*	MFLUCC 17-2073	MT310633	MT214588	MT226701	MT394646	MT394702
*N.heveae*	MFLUCC 17-1983	MH590693	MH590689	MH590691	–	–
*N.lenispora*	GZCC 16-0020 T	–	KX791431	–	–	–
*N.leucaenae*	MFLUCC 18-1544	MK347767	MK347984	MK347874	MK360067	MK434876
*N.leucaenae*	MFLUCC 17-0927	MK347733	MK347950	MK347841	MK360066	MK434896
*N.lignicola*	MUT 5008	MN556318	MN556320	MN556308	MN605895	MN605915
*N.lignicola*	MUT 5373	KU314953	MN556321	KU314954	MN605896	MN605916
*N.lignicola*	MUT 4904	KT699129	MN556319	MN556307	MN605894	MN605914
*N.magnoliae*	MFLU 18-1022	MK801232	MK801230	MK801231	MK834373	–
*N.peltophora*	MFLUCC 21-0071	MZ567051	MZ567206	MZ567207	MZ605441	MZ605442
*N.sedimenticola*	CGMCC 3.22470	OQ798949	OQ758144		OQ809046	OQ809008
*N.sedimenticola*	CGMCC 3.22468 T	OQ798948	OQ758143		OQ809045	OQ809007
*N.solani*	KT3264 T	LC195218	LC195209	LC195206	LC195212	–
*N.solani*	KT3265 T	LC195219	LC195210	LC195207	LC195213	LC195216
*N.thailandica*	MFLUCC 18-0721	OL703581	OL457704	OL764415	OM505028	ON502386
*Occultibambusabambusae*	MFLUCC 11-0394	KU940124	KU863113	–	KU940194	KU940171
*O.bambusae*	MFLUCC 13-0855	KU940123	KU863112	KU872116	KU940193	KU940170
*Pseudoneoconiothyriumrosae*	MFLUCC 15-0052 T	MG828922	MG829032	MG829138	–	–
*P.euonymi*	CBS 143426 T	MH107915	MH107961	–	–	MH108007
*P.euonymi*	GLMC 1544	MT153733	MT156304	–	–	–
***Pseudoroussoellabidenticola* sp. nov.**	**MFLUCC 24-0273**	**PQ226192**	**PQ226195**	**PQ226198**	**PQ240622**	**PQ240625**
*Ps.chromolaenae*	MFLUCC 17-1492 T	MT214345	MT214439	MT214393	MT235769	–
*Ps.elaeicola*	MFLUCC 15-0276a T	MT153733	MT156304	–	–	–
*Ps.elaeicola*	MFLUCC 15-0276b	MH742330	MH742327	–	–	–
*Ps.elaeicola*	MFLUCC 17-1483	MT214348	MT214442	–	MT235772	MT235808
*Pararoussoellamangrovei*	MFLUCC 17-1542	MH025951	MH023318	–	MH028246	–
*P.mukdahanensi*	HKAS 101766	MH453489	MH453485	–	MH453478	MH453482
*P.rosarum*	MFLUCC 17-0796 T	MG828939	MG829048	NG_061294	MG829224	MH028250
*Roussoellaarundinacea*	CPC 35554	MT223838	MT223928	–	MT223723	–
*R.bambusarum*	GMB0316(HT)	ON479891	ON479892	–	ON505015	ON505011
*R.bambusarum*	GMB0390	ON505055	ON505051	–	ON505017	ON505012
*R.chiangraina*	MFLUCC 10-0556 T	KJ474828	KJ474840	–	KJ474849	KJ474857
*R.doimaesalongensis*	MFLUCC 14-0584 T	KY026584	KY000659	–	KY651249	KY678394
*R.hysterioides*	CBS 546.94 T	KF443405	KF443381	AB524480	KF443399	KF443392
*R.intermedia*	CBS 170.96	KF443407	KF443382	KF443390	KF443398	KF443394
*R.japanensis*	MAFF 239636 T	KJ474829	AB524621	–	AB539114	AB539101
*R.kunmingensis*	HKAS 101773	MH453491	MH453487	–	MH453480	MH453484
*R.margidorensis*	MUT 5329 T	KU314944	MN556322	MN556309	MN605897	MN605917
*R.mediterranea*	MUT 5306	KU255054	MN556323	MN556310	MN605898	MN605918
*R.mexicana*	CPC25355 T	KT950848	KT950862	–	–	–
*R.neopustulans*	MFLUCC 11-0609 T	KJ474833	KJ474841	–	KJ474850	–
*R.neopustulans*	MFLUCC 12-0003 T	KU940130	KU863119	KU872122	–	–
*R.nitidula*	MFLUCC 11-0182 T	KJ474835	KJ474843	–	KJ474852	KJ474859
*R.nitidula*	MFLUCC 11-0634 T	KJ474834	KJ474842	–	KJ474851	KJ474858
*R.padinae*	MUT 5341	KU158153	MN556325	–	MN605900	MN605920
*R.padinae*	MUT 5365	KU158170	MN556326	KU158179	MN605901	MN605921
*R.padinae*	MUT 5503	KU314993	MN556327	MN556312	MN605902	MN605922
*R.pseudohysterioides*	MFLUCC 13-0852 T	KU940131	KU863120	–	KU940198	–
*R.pustulans*	KT 1709	–	AB524623	AB524482	AB539116	AB539103
*R.scabrispora*	MFLUCC 11-0624	KJ474836	KJ474844	–	KJ474853	KJ474860
*R.siamensis*	GMB0317	ON4617749	ON461896	–	ON505014	ON505010
*R.siamensis*	GMB0391	ON505054	ON505053	–	ON505018	ON505013
*R.tosaensis*	KT 1659	–	AB524625	AB524484	AB539117	AB539104
*R.tuberculata*	MFLUCC 13-0854 T	KU940132	KU863121	–	–	–
*R.verrucispora*	CBS 125434 T	KJ474832	–	–	–	–
*R.yunnanensis*	HKAS 101762 T	MH453492	MH453488	–	MH453481	–
*R.yunnanensis*	MFLUCC 12-0005 T	KJ739604	KJ474847	KJ739608	KJ474855	KJ474862
*Xenoroussoellatriseptata*	MFLUCC 17-1438	MT214343	MT214437	MT214391	MT235767	MT235804
*X.triseptata*	KNUF-20-NI009	LC719282	LC719283	LC723530	LC723531	LC723532

* Remarks: The letter T denotes ex-type isolates. The newly generated sequences, new species and synonymized isolates are indicated in bold font.

### ﻿Sequence alignment and phylogenetic analyses

Based on the sequence data of recent publications (De Silva et al. 2020; Li et al. 2023) and BLAST search results, reference taxa were selected, and phylogenetic analyses were conducted using the combined LSU, SSU, ITS, *tef1-α* and *rpb2* sequence data. Sequence alignments were made with the MAFFT v. 7 online tool (http://mafft.cbrc.jp/alignment/server; 2016). Alignments were improved where necessary, and composite sequence alignments were obtained using MEGA v. 6.0.

RAxML and Bayesian analyses were carried out on the CIPRESS Science Gateway Portal (http://www.phylo.org) (Miller et al. 2010). Maximum likelihood analysis was performed by RAxML-HPC v.8 (Stamatakis 2014) with rapid bootstrap analysis, followed by 1000 bootstrap replicates and the GTRGAMMA substitution model. MrBayes was used to perform BI analysis on XSEDE 3.2.7 (Ronquist et al. 2012), with tree samples taken at every 1000^th^ generation during the 5,000,000-generation run of four concurrent Markov chains. The first 25% of the trees were removed as part of the burn-in phase, and calculations for the Posterior Probability were made for the remaining 75% of the trees (PP) (Rannala and Yang 1996; Zhaxybayeva and Gogarten 2002). The phylogenetic tree was displayed using Fig. Tree v1.4.0 (Rambaut 2012) and was modified in Microsoft Office PowerPoint v. 2013.

### ﻿Preliminary screening for antibacterial activity

Preliminary screening for antibacterial activity was carried out following the methods of Htet et al. (2024). Antibacterial discs of ampicillin were used as a positive control for screening (Alam et al. 2019). Antibacterial activity against gram positive bacteria (*Bacillussubtilis*-TISTR 1248 and *Staphylococcusaureus*-TISTR Y4b), and gram-negative bacteria (*Escherichiacoli* TISTR 527) were investigated using the agar plug diffusion method (Balouiri et al. 2016). Bacteria test organisms were obtained from Scientific and Technological Instrument Center, Mae Fah Luang University. Bacterial test organisms were sub cultured and grown on Nutrient Agar (NA) for 24 hours. After 24 hours of inoculation, 2–3 loops of the bacterial test organisms were transferred to the nutrient broth and incubated for 24 hours in a shaking incubator. Prior to adding microbial suspensions to the sterile Mueller-Hinton agar media, cell counts were performed on the suspensions (6.7 × 10^5^ cells/mL), as detailed by Mapook et al. (2020). Fungal mycelium plugs from our isolates were transferred to a solid medium plate and allowed to grow at room temperature for 24–48 hours. Inhibition zones were measured and compared to the positive control.

## ﻿Results

### ﻿Phylogenetic analyses

**Figure 1.**
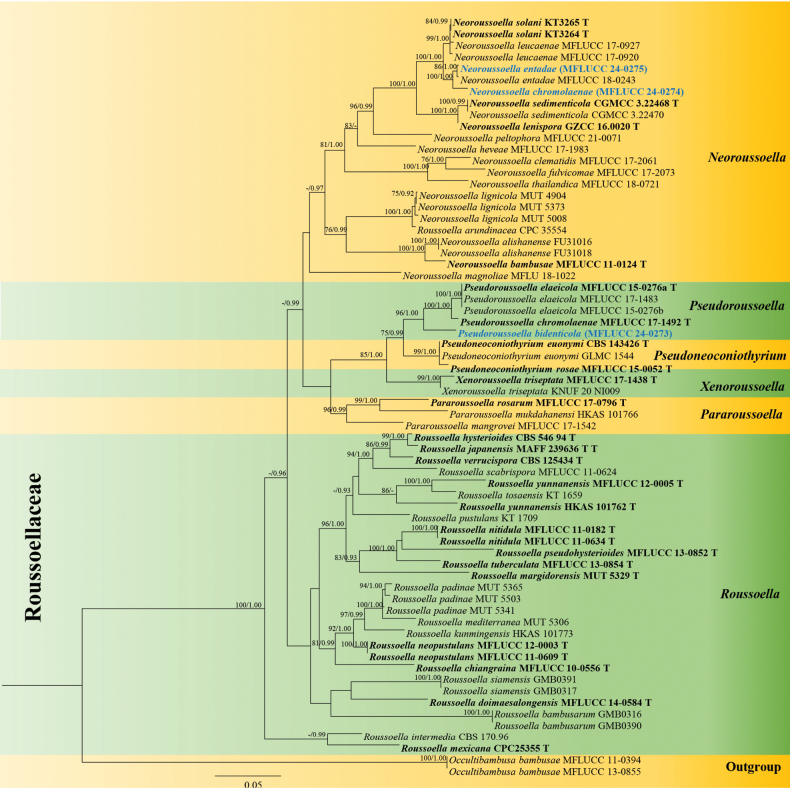
Phylogram generated from maximum likelihood analysis based on the combined dataset of LSU, SSU, ITS, *tef1-α* and *rpb2* sequence data. Bootstrap support values for ML equal to or greater than 75% and BYPP equal to or greater than 0.90 are given at the nodes. Newly generated sequences are in blue and type species are in bold.

The combined LSU, SSU, ITS*tef1-α*, and *rpb2* sequence dataset comprises 66 taxa with *Occultibambusabambusae* strains (MFLUC 13-0855 and MFLUCC 11-0394) as the outgroup taxa. Maximum likelihood (ML) analyses and Bayesian Inference (BI) of the combined dataset were performed to determine the placement of our new isolates. Tree topologies of ML and BI criteria were similar to earlier investigations (De Silva et al. 2020; Li et al. 2023). The best-scoring RAxML tree with a final likelihood value of -28736.822321 is shown in Fig. 1. RAxML analysis yielded 1578 distinct alignment patterns, with 29.39% of undetermined characters or gaps. Estimated base frequencies were as follows: A = 0.245746, C = 0.258383, G = 0.266559, T = 0.229312; substitution rates: AC = 1.714222, AG = 4.957697, AT = 1.884927, CG = 1.345111, CT = 9.562411, GT = 1.000000; gamma distribution shape parameter *α* = 0.166563. In our phylogenetic analysis, *Neoroussoellachromolaenae* sp. nov. (MFLUCC 24-0274) clustered with *Neoroussoellaentadae* (MFLUCC 18-0243 and MFLUCC 24-0275) with 100% ML and 1.00 BYPP support. Furthermore, our isolate, *Pseudoroussoellabidenticola* sp. nov. (MFLUCC 24-0273) formed a branch separated from *Ps.elaeicola* (MFLUCC 17-1483, MFLUCC 15-0276a, MFLUCC 15-0276b), and *Ps.chromolaenae* (MFLUCC 17-1492) with 96% ML and 1.00 BYPP, respectively.

### ﻿Taxonomy

#### ﻿ Neoroussoella chromolaenae

Taxon classificationFungiPleosporalesRoussoellaceae

Z.H. Htet, A. Mapook & K. D. Hyde sp. nov.

4EF5EA92-5E50-5E22-8BE2-AA307EF9C9C8

Index Fungorum: IF902613

Facesoffungi Number: FoF16402

Fig. 2

##### Etymology.

Name reflects the host plant *Chromolaenaodorata*, from which this species was isolated.

##### Holotype.

MFLU 24-0264.

##### Description.

***Saprobic*** on the dead stems of *Chromolaenaodorata* (Asteraceae). ***Sexual morph***: Undetermined. ***Asexual morph***: Coelomycetous. ***Conidiomata*** 70–150 × 120–150 µm (av. 85 × 138 µm, n = 5), pycnidial, solitary, uniloculate, immersed, ostiolate. ***Ostiole*** papillate. ***Peridium*** 10–20 µm wide, comprising 2–3 layers of brown cells of ***textura angularis***. ***Conidiophores*** reduced to conidiogenous cells. ***Conidiogenous cells*** 3–5 × 2–3.5 µm (av. 3 × 3 µm, n = 10), phialidic, ampulliform to cylindrical, hyaline. ***Conidia*** 3–6 × 2–4 μm (av. 4.4 × 3.1 μm, n = 20), hyaline, oblong to slightly ellipsoid, aseptate, with small guttules.

##### Culture characteristics.

Conidia germinating on MEA within 24 hours, reaching 22 mm after 10 days at 27 °C, irregular, curled margin, brown in the middle and becoming pale brown on the outer parts of the culture, wrinkled on the surface; wrinkle, pale brown to brown in reverse.

**Figure 2.**
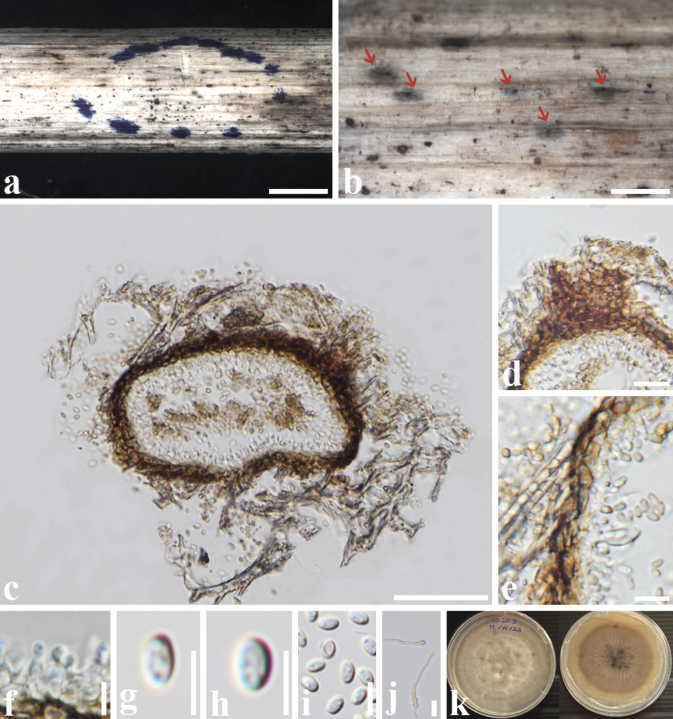
*Neoroussoellachromolaenae* (MFLU 24-0264, holotype) **a, b** Conidiomata on the substrate **c** a section through conidioma **d** ostiole **e** peridium **f** conidia and conidiogenous cells **g–i** conidia **j** germinating conidia **k** culture on the MEA. Scale bars: 500 µm (**a, b**); 100 µm (**c**); 20 µm (**d, e**); 10 µm (**e–j**).

##### Material examined.

Thailand • Chiang Rai Province, Doi Pui, 19°48'51"N, 99°52'1"E, on dead stems of *Chromolaenaodorata* (Asteraceae), 14 March 2023, Zin Hnin Htet (CO-DP-3, MFLU 24-0264, holotype); ex-type culture MFLUCC 24-0274.

##### Notes.

In a megablast search of GenBank, the closest match for the ITS sequence of our isolate was fungal sp. isolate NFC-3 (MG189955) with 99.47% similarity. The closest match for the LSU region was *N.solani* CBS 141288 (MH878207) with 100% similarity, and the closest match for the SSU region was *N.bambusae* strain GMB1295 (OM764650) with 93.99% similarity. Additionally, the closet matches for the *tef1-α* and *rpb2* gene regions were *Neoroussoellaentadae* strain MFLUCC 18-0243 (MK360065) and *N.entadae* strain MFLUCC 17-0920 (MK434898) with 99.45% and 99.77% similarities, respectively.

Based on the multi-locus phylogeny (Fig. 1), our isolate (MFLUCC 24-0274) formed a separate branch from *N.entadae* with 100% ML and 1.00 BYPP. A comparative analysis of base pair differences between *Neoroussoellachromolaenae* (MFLUCC 24-0274) and *Neoroussoellaentadae* (MFLUCC 18-0243) revealed variations in ITS (0.6% - 3/476), LSU (0.1% - 1/838), SSU (1.9% - 14/717), *tef1-α* (0.5% - 5/902), and *rpb2* (2.0% - 18/885) without gaps, respectively. Morphologically, our collection is similar to *N.entadae* (MFLUCC 17–0920) in having solitary, unilocular, ostiolate, phialidic, ampulliform to cylindrical, hyaline conidiogenous cells, and oblong to ellipsoidal, hyaline conidia (Jayasiri et al. 2019). However, our species differs from *N.entadae* (MFLUCC 17–0920) in having smaller conidiomata (70–150 × 120–150 µm vs. 127–192 × 161–190 µm), slightly wider conidiogenous cells (2–3.5 µm vs. 0.7–1.8 µm) and larger conidia size (3–6 × 2–4 μm vs. 3–4 × 1.7–1.9 μm). Therefore, *N.chromolaenae* is described here as a new species based on phylogeny and morphological evidence. Synopsis of the asexual morph of *Neoroussoella* species is also provided in Table 3.

**Table 3.** Synopsis of asexual morph species in *Neoroussoella*.

Species	Conidiomata (µm)	Conidiogenous cells (µm)	Conidia (µm)	References
*Neoroussoellaalishanense* (FU31016)	130–140, 210–225	8–14 × 2–3	3–4 × 2–3	Karunarathna et al. (2019)
*Neoroussoellabambusae* (MFLUCC 11-0124)	200–430 × 300–420	8–13.5 × 1.5–3	3–4 × 1.5–2	Liu et al. (2014)
*Neoroussoellachromolaenae* (MFLUCC 24-0274)	70–150 × 120–150	3–5 × 2–3.5	3–6 × 2–4	This study
*Neoroussoellaentadae* (MFLUCC 17–0920)	127–192 × 161–190	3.5–5.6 × 0.7–1.8	3–4 × 1.7–1.9	Jayasiri et al. (2019)
*Neoroussoellaentadae* (MFLUCC 24-0275)	70–120 × 100–150	3–5 × 1–3	3–5 × 2–4	This study
*Neoroussoellaheveae* (MFLUCC 17-0338)	90–130, 115–180	3–7 × 2–5	2.5–5 × 2–4	Phookamsak et al. (2019)
*Neoroussoellaleucaenae* (MFLUCC 18–1544)	135–175 × 120–180	5.5–9 × 3–4	3.5–4.5 × 1.9–2.6	Jayasiri et al. (2019)
*Neoroussoellapeltophora* (MFLUCC 21-0071)	165–224 × 144–178	1–3.5 × 6.5–8	2.0–3.0 × 3.0–4.0	Pasouvang et al. (2022)
*Neoroussoellasolani* (CPC 26331)	To 150	4–6 × 3–4	4.5–5 × 2–3	Crous et al. (2016)

#### ﻿ Neoroussoella entadae

Taxon classificationFungiPleosporalesRoussoellaceae

Jayasiri, E.B.G. Jones & K.D. Hyde, Mycosphere 10(1): 105 (2019)

1CAEDF93-E94B-5388-B4E3-D88B954D009B

Index Fungorum: IF555568

Facesoffungi Number: FoF05275

Fig. 3

##### Description.

***Saprobic*** on the dead stems of *Chromolaenaodorata* (Asteraceae). ***Sexual morph***: Undetermined. ***Asexual morph***: Coelomycetous. ***Conidiomata*** 70–120 × 100–150 µm (av. 95 × 110 µm, n = 5), pycnidial, solitary, globose to subglobose, uniloculate, immersed to semi-immersed, ostiolate. ***Ostiole*** 30–35 µm wide, with small papillate. ***Peridium*** 10–20 µm wide, comprising 2–3 layers of brown cells of ***textura angularis***. ***Conidiophores*** reduced to conidiogenous cells. ***Conidiogenous cells*** 3–5 × 1–3 µm (av. 3.8 × 2.1 µm, n = 10), phialidic, ampulliform to cylindrical, hyaline. ***Conidia*** 3–5 × 2–4 μm (av. 3.8 × 2 μm, n = 20), hyaline, becoming pale brown when gathering, oblong to ovoid, aseptate, with small guttules.

##### Culture characteristics.

Conidia germinating on PDA within 24 hours, reaching 24 mm after 7 days at 27 °C, irregular, entire, yellowish-brown, slightly wrinkled on the surface; pale brown to brown in reverse.

**Figure 3.**
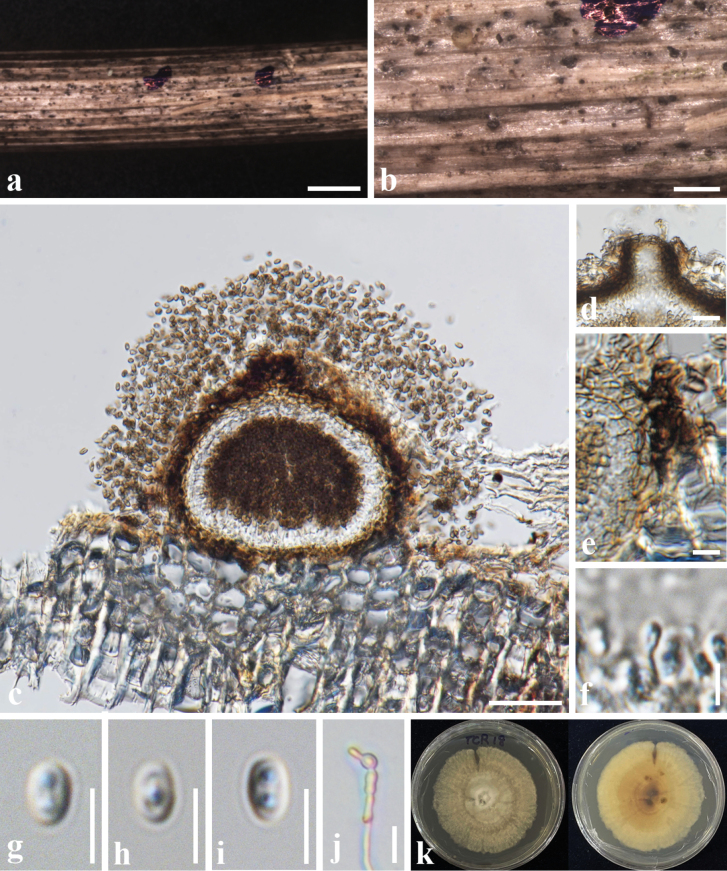
*Neoroussoellaentadae* (MFLU 24-0265, new host record) **a, b** Conidiomata on the substrate **c** a section through conidioma **d** ostiole **e** peridium **f** conidia and conidiogenous cells **g–i** conidia **j** a germinating conidium **k** culture on the MEA. Scale bars: 500 µm (**a**); 200 µm (**b**); 50 µm (**c**); 20 µm (**d, e**); 5 µm (**f, g, h, i, j**).

##### Material examined.

Thailand • Chiang Rai Province, Thoeng district, on dead stems of *Chromolaenaodorata* (Asteraceae), 24 Jan 2022, A. Mapook (TCR18, MFLU 24-0265, new host record); living culture MFLUCC 24-0275.

##### Known host distribution.

*Entadaphaseoloides* (Fabaceae), *Leucaena* sp. (Fabaceae) (Jayasiri et al. 2019).

##### Notes.

In a BLASTn search of GenBank, the closest match for the ITS sequence of our isolate was *N.solani* strain MnF107 (OQ704272) with 99.83% similarity. The closest match for the LSU region was *Roussoella* sp. strain HF3S53 (OP179275) with 99.77% similarity, and the closest match for the SSU region was Pleosporales sp. IRB20-2 (AB195632) with 100% similarity. The closest match for the *tef1-α* and *rpb2* gene region was *Neoroussoellaentadae* strain MFLUCC 18-0243 (MK434866) with 99.78% and 99.53% similarity, respectively.

When we compared the morphology, our isolate is similar to *N.entadae* (MFLUCC 17–0920) in having solitary, unilocular, ostiolate conidiomata, phialidic, ampulliform to cylindrical, hyaline conidiogenous cells, and oblong to ovoid, hyaline conidia with size (3–5 × 2–4 μm vs 3–4 × 1.7–1.9 μm). However, our isolate differs from *N.entadae* (MFLUCC 17–0920) in having smaller conidiomata (70–120 × 100–150 µm vs. 127–192 × 161–190 µm), slightly wider conidiogenous cells (3–5 × 1–3 µm vs. 3.5–5.6 × 0.7–1.8 µm) (Table 3).

Based on the multi-locus phylogeny (Fig. 1), our isolate MFLUCC 24-0275 clustered in the same clade with *N.entadae* (MFLUCC 18-0243). Moreover, there is no significant base pair difference between MFLUCC 24-0275 and *N.entadae* (MFLUCC 18-0243). Therefore, we reported *N.entadae* as the first occurrence on *C.odorata* (Asteraceae) based on morphology and multigene phylogeny.

#### ﻿ Pseudoroussoella bidenticola

Taxon classificationFungiPleosporalesRoussoellaceae

Z.H. Htet, A. Mapook & K. D. Hyde sp. nov.

B8C83E96-AA47-515B-916B-D5987D35EA92

Index Fungorum: IF902614

Facesoffungi Number: FoF16403

Fig. 4

##### Etymology.

Name reflects the host plant *Bidenspilosa*, from which this species was isolated.

##### Holotype.

MFLU 24-0266.

##### Description.

***Saprobic*** on dead stems of *Bidenspilosa*. ***Sexual morph***: Undetermined. ***Asexual morph***: Coelomycetous. ***Conidiomata*** 120–150 × 150–180 µm (av. 126 × 173 µm, n = 5), pycnidial, solitary, immersed to semi-immersed, uni-loculate, brown, globose to subglobose, dark fruiting bodies on the host substrate, without an ostiole. ***Peridium*** 10–20 µm wide, comprising 2–3 layers of yellowish brown to brown cells of ***textura angularis***. ***Conidiophores*** reduced to conidiogenous cells. ***Conidiogenous cells*** 1–2 μm long, holoblastic, short, globose to subglobose, hyaline and unbranched. ***Conidia*** 5–7.5 × 4–5.5 μm (av. 6 × 4.8 µm, n = 20), globose to subglobose, brown to reddish brown, aseptate, thick-walled with a guttule.

**Figure 4.**
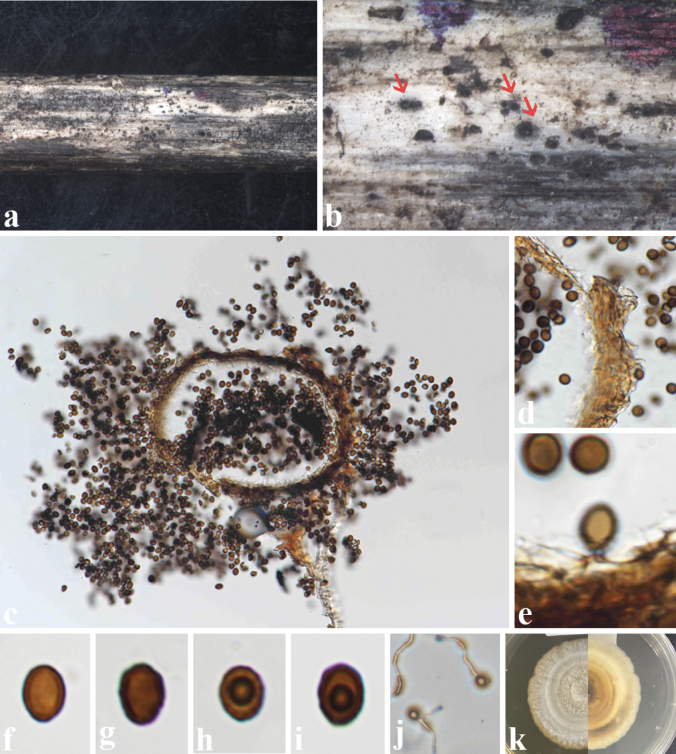
*Pseudoroussoellabidenticola* (MFLU 24-0266, holotype) **a**, **b** Conidiomata on the substrate **c** a section through a conidioma **d** peridium **e** conidia and conidiogenous cell **f**–**i** conidia **j** germinating conidia **k** culture on the MEA. Scale bars: 500 µm (**a, b**); 100 µm (**c**); 10 µm (**d**); 5 µm (**e–i**).

##### Culture characteristics.

Conidia germinating on MEA within 24 hours, reaching 27 mm after 10 days at 27 °C, irregular, entire, concentric, opaque, flat, white to pale brown on the surface; concentric, creamy to pale brown in reverse.

##### Material examined.

Thailand • Chiang Rai Province, Doi Pui, 19°48'51"N, 99°52'1"E, on dead stems of *Bidenspilosa* (Asteraceae), 14 March 2023, Zin Hnin Htet (BP-DP-11, MFLU 24-0266, holotype); ex-type culture MFLUCC 24-0273.

##### Notes.

In a BLASTn search of GenBank, the closest match for the ITS sequence of our isolate was *Roussoellaelaeicola* strain MFLUCC 15-0276b (MH742330) with 94.57% similarity. The closest match for the LSU region was *Pseudoroussoellachromolaenae* isolate MFLUCC 17-2062 (MT394704) with 92.95% similarity, and the closest match for the SSU region was *Parathyridariatyrrhenica* MUT<ITA>:5371 (KU314952) with 99.16% similarity. Additionally, the closest matches for the *tef1-α* and *rpb2* gene regions were *Pseudoroussoellaelaeicola* culture MFLUCC:17-1483 (MT235772) and *Roussoella* sp. strain GMB1153 (OM755588) with 97.08% and 98.27% similarity, respectively.

*Pseudoroussoellaelaeicola* (MFLUCC 17-1483 and MFLUCC 17–2086) was found as a sexual morph in nature (Phookamsak et al. 2019, Mapook et al. 2020); hence, we were unable to directly compare their morphology with our isolate. However, based on comparing the morphology of *Pseudorousoellabidenticola* (MFLUCC 24-0273) and *Ps.chromolaenae* (MFLUCC 17-1492), our species differs from *Ps.chromolaenae* (MFLUCC 17-1492) in having immersed to semi-immersed, globose to subglobose, brown, conidiomata without ostiole, smaller-sized (120–150 × 150–180 µm vs 130–175(–230) × 160–230 µm), holoblastic, globose to subglobose conidiogenous cells, and brown to reddish brown, globose to subglobose conidia with guttules, while *Ps.chromolaenae* (MFLUCC 17-1492) displays superficial, globose to obpyriform, yellowish brown to brown conidiomata with a central ostiole, annellidic, ampulliform to oblong conidiogenous cells, and oblong to oval, conidia that are pale brown to light brown when immature, becoming yellowish brown to reddish brown when mature (Table 4).

**Table 4.** Synopsis of sexual and asexual morph of *Chromolaenicola* species.

Species	Conidiomata (µm)	Conidiogenous cells (µm)	Conidia (µm)	References
*Pseudoroussoellachromolaenae* (MFLUCC 17-1492)	130–175(–230) × 160–230	–	5.5–7 × 3.5–5	Mapook et al. (2020)
*Pseudoroussoellabidenticola* (MFLUCC 24-0273)	120–150 × 150–180	1–2	5–7.5 × 4–5.5	This study
**Species**	**Ascomata (µm)**	**Asci (µm)**	**Ascospores (µm)**	**References**
***Pseudoroussoellaelaeicola***	225–475 × 240–400	95–135 × 6–8.5	10–14 × 4.5–6	Mapook et al. (2020)

Based on the multi-locus phylogeny (Fig. 1), our isolate (MFLUCC 24-0273) formed a separate branch related to *Pseudoroussoella* species with 96% ML and 1.00 BYPP. When comparing base pair differences between *Ps.bidenticola* (MFLUCC 24-0273) and *Ps.chromolaenae* (MFLUCC 17-1492), variations were observed in ITS (3.6% - 23/469), LSU (0.6% - 5/799), SSU (0.6% - 4/630), *tef1-α* (2.6% - 24/891), without gaps. Therefore, we introduced our collection (MFLUCC 24-0273) as a new species based on morphology and multigene phylogeny. Moreover, this is also the first record of *Pseudoroussoella* species from *Bidenspilosa* (Asteraceae).

### ﻿Preliminary screening for antibacterial activity

In our study, we explored the antibacterial activities of our three isolates against *Bacillussubtilis*, *Escherichiacolicoli*, and *Staphylococcusaureus*. *Neoroussoellachromolaenae* (MFLUCC 24-0274), *N.endatae* (MFLUCC 24-0275), *Pseudoroussoellabidenticola* (MFLUCC 24-0273) exhibited antibacterial activity against all three test organisms. For *B.subtilis*, *N.chromolaenae* (MFLUCC 24-0274), *N.entadae* (MFLUCC 24-0275), and *Ps.bidenticola* (MFLUCC 24-0273) exhibited partial inhibition. Against *E.coli*, *N.chromolaenae* (MFLUCC 24-0274), *N.entadae* (MFLUCC 24-0275), and *Ps.bidenticola* (MFLUCC 24-0273) demonstrated clear inhibition. For *S.aureus*, *N.chromolaenae* (MFLUCC 24-0274) showed the most significant inhibition, followed by *N.entadae* (MFLUCC 24-0275) and *Ps.bidenticola* (MFLUCC 24-0273), with clear inhibition observed. However, none of these fungal species showed a wider inhibition zone than the positive control, ampicillin (20 mm for *B.subtilis*, 50 mm for *E.coli*, 40 mm for *S.aureus*). The measurements of clear inhibition zones produced by our new isolates are provided in Table 5.

**Table 5.** Preliminary antibacterial activity result of this study.

Species	Zone of inhibition (mm); Ampicillin (+)		
	*Bacillussubtilis*	*Escherichiacoli*	*Staphylococcusaureus*
*Neoroussoellachromolaenae* (MFLUCC 24-0274)	16 mm inhibition	11 mm inhibition	20 mm inhibition
*N.entadae* (MFLUCC 24-0275)	13 mm inhibition	17 mm inhibition	14 mm inhibition
*Pseudoroussoellabidenticola* (MFLUCC 24-0273)	18 mm inhibition	12 mm inhibition	13 mm inhibition

Abbreviation: Positive control (+).

## ﻿Discussion

Our research in northern Thailand unveiled the introduction of two novel species and one new host record within the Roussoellaceae. This classification was determined through a combination of morphological analyses and a multigene phylogeny approach, adhering to the recommendations outlined by Jeewon and Hyde(2016). Mapook et al. (2020) established *Pseudoroussoella* to accommodate *Ps.chromolaenae* and *Ps.elaeicola*. Interestingly, our study revealed a third *Pseudoroussoella* strain on the dead stems of *Bidenspilosa* (Asteraceae). In our phylogenetic analyses, our isolate (MFLUCC 24-0273) formed a basal lineage to other strains (MFLUCC 17-1483; MFLUCC 15-0276a; MFLUCC 15-0276b and MFLUCC 17-1492). Our species is morphologically similar to *Ps.chromolaenae* and has a significant base pairs difference between the two, and herein, we introduced our isolate as a new species. Moreover, we found two isolates of *Neoroussoella* on the dead stems of *C.odorata* (Asteraceae). Based on the morphological similarity and multigene phylogeny, we identified *N.chromolaenae* as a new species and *N.entadae* as the first occurrence on *Chromolaenaodorata*.

Following the preliminary screening for antibacterial activity, all species examined in our study demonstrated potential antibacterial properties. In a prior examination of *Pseudoroussoella* species, *Ps.chromolaena* exhibited no inhibition on *B.subtilis*, *E.coli*, and *M.plumbeus*, while *Ps.elaeicola* displayed a 10 mm inhibition zone against *E.coli* (Mapook et al. 2020). Our isolate (MFLUCC 24-0273) exhibited inhibition on all tested organisms, with inhibition zones measuring 18 mm (*B.subtilis*), 12 mm (*E.coli*), and 13 mm (*S.aureus*), respectively. These results highlight our new species, *Pseodoroussoellabidenticola*, as a particularly promising organism for further research. Additionally, *Neoroussoellachromolaenae* (MFLUCC 24-0274) and *N.entadae* (MFLUCC 24-0275) demonstrated inhibitory effects against all test organisms, and specific measurements of inhibition zones are shown in Table 4. Across the three observed species, all species exhibited antibacterial activity. The outcomes of our investigation suggest that this fungal group possesses antibacterial potential, presenting a source for the exploration of novel bioactive compounds. These findings also highlight the potential of the Roussoellaceae family for antibacterial compound discovery, a field that remains relatively underexplored.

## Supplementary Material

XML Treatment for Neoroussoella chromolaenae

XML Treatment for Neoroussoella entadae

XML Treatment for Pseudoroussoella bidenticola

## ﻿Additional information

### Author ORCIDs

Zin Hnin Htet https://orcid.org/0000-0002-6652-6975

Kevin D. Hyde https://orcid.org/0000-0002-2191-0762

Fatimah O. Alotibi https://orcid.org/0000-0003-3629-5755

Thilini K. W. Chethana https://orcid.org/0000-0002-5816-9269

Ausana Mapook https://orcid.org/0000-0001-7929-2429

### Data availability

All of the data that support the findings of this study are available in the main text.
